# Novel Medication Ganaxolone for Seizure Disorders

**DOI:** 10.15190/d.2025.7

**Published:** 2025-06-30

**Authors:** Selia Chowdhury, Samia Chowdhury

**Affiliations:** ^1^Dhaka Medical College, Dhaka, Bangladesh; ^2^Sylhet MAG Osmani Medical College, Sylhet, Bangladesh

**Keywords:** Ganaxolone, seizure, epilepsy, anti-epileptic.

## Abstract

Ganaxolone, a neuroactive steroid that increases GABAergic inhibition, has been tested in many trials for the resolution of different epileptic disorders. Based on these, our study implemented a systematic review to evaluate the general benefit of ganaxolone for seizures. Pubmed, google scholar, European PubMed Central, Cochrane Library, and U.S. National Library of Medicine Clinical Trials were searched for relevant randomized controlled trials (RCTs) up to March 2023 and 12 RCTs were included in this review. We included 1337 patients from 12 RCTs to evaluate the efficacy and safety of ganaxolone. As results showed, across all trials, 26.3% of participants reported >50% reduction from baseline in seizure frequency. The analysis revealed that the nervous system was the most impacted, with dizziness, somnolence, headache, and seizures being the most frequently reported treatment-related adverse events (TRAEs). The results of these trials suggest that ganaxolone may be effective in reducing seizure frequency in patients with various types of epilepsy, including CDKL5 deficiency disorder, Lennox-Gastaut syndrome, and Protocadherin 19. However, further studies are needed to determine its safety and efficacy in a larger patient population.

## INTRODUCTION

Seizure disorders are a group of neurological disorders that are characterized by recurrent seizures, which can lead to significant disability and reduced quality of life for those affected. Ganaxolone is a new medication designed to treat such disorders. Ganaxolone is a synthetic analogue of the allopregnanolone, which is a neuroactive steroid gamma-aminobutyric acid (GABA) A receptor modulator. Ganaxolone is not hormonally active because the 3beta-methyl substituent inhibits its oxidation and metabolism on the 3alpha-hydroxy moiety, hence avoiding the associated adverse effects^^1^^. Unlike benzodiazepines, which only bind to GABA-A receptors, ganaxolone also binds to GABA-A receptors containing the d-subunits^^2^^. It has been shown to effectively control seizures in multiple animal models and has recently been approved by the FDA in the US for the treatment of seizures associated with cyclin-dependent kinase-like 5 (CDKL5) deficiency disorder (CDD) in patients 2 years of age and older^^3^^.

CDD is a relatively common cause of genetic childhood-onset developmental and epileptic encephalopathy. Ganaxolone is the first FDA-approved treatment for CDD-associated seizures^^4^^. It has shown promise as a potential treatment for various other types of seizure disorders, including refractory epilepsy and status epilepticus. The medication is currently being evaluated for the treatment of seizures associated with tuberous sclerosis complex (TSC) and status epilepticus and several other seizure disorders. However, there is a need for a comprehensive and systematic review of the available evidence to determine the efficacy and safety of ganaxolone in the treatment of different types of seizure disorders. In this systematic review we explore several randomized controlled trials to estimate the safety and efficacy of ganaxolone due to variability among studies.

## METHODS

**Figure 1:**
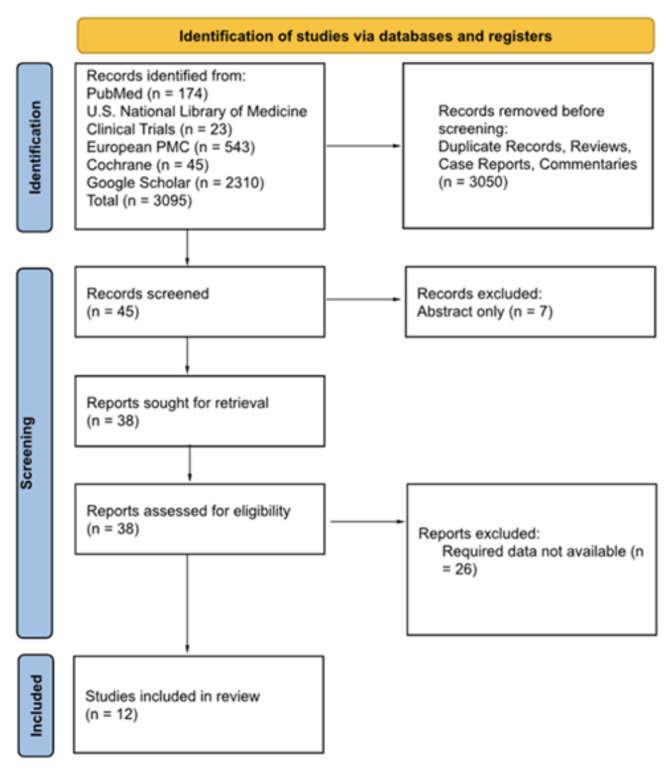
PRISMA flow diagram showing selection process of the studies included in this review.

A systematic review was performed to identify randomized controlled trials (RCTs) that investigated the effectiveness of ganaxolone therapy as an antiepileptic drug. The search was conducted from 1997 to February 2023, using various databases such as the National Institute of Health, US National Library of Medicine Clinical Trials, PubMed, European PubMed Central, and the Cochrane Library. Additionally, conference abstracts from the Cochrane database and drug information from the FDA label were examined. This review only included articles published in English that evaluated the efficacy, safety, and tolerability of ganaxolone as an antiepileptic drug. Excluded from this review were non-primary literature, such as reviews, meta-analyses, and secondary analyses. Furthermore, conference proceedings were not included as they pertained to clinical trials, which were already extensively discussed (Figure 1).

## RESULTS

### Trials and Participants Characteristics

A systematic search method was employed, which yielded 3095 results across five selected databases as mentioned in the methods section. After screening

titles and abstracts, 38 studies were selected for full-text screening, of which 12 trials were included in this review after screening and excluding the results that didn’t report efficacy, tolerability, and safety of ganaxolone (Figure 1). No time or language filters were used during the search.

A total of 1337 participants were included in all the trials, with a mean age (SD) of 25.4 (17.1). The majority of the participants were female (60.2%) and Caucasian (88.7%). Eight trials were designed as open-label^^5-11^^, seven as double-blind^^7,12-16^^, and six trials were placebo-controlled^^7,12-16^^. While^^8-10,13-15^^ conducted a phase 2 trial, three conducted a phase 3 trial^^7,11,16^^, and it should be noted that some studies did not report their trial phase^^5,6,12^^. The doses of ganaxolone varied across all studies, but five studies reported administering a maximum dose of 1800mg/day^^7,9,11,14,16^^ (Table 1). In their research studies, Pieribone et al.^^6^^ and Sperling et al. ^^13^^ recorded the baseline seizure frequency of participants in the ganaxolone and placebo groups. The average number of seizures per day^^6^^ was found to be mean (SD) 7.42 (11.6), and the average number of seizures per week^^13^^ was 6.5 (11.3) for the

**Table 1:** Description of Trials and Characteristics of Trial Participants *^*a*^* *: Terminated because ganaxolone missed its primary endpoint in the double-blind portion of the 1042-0603 NCT01963208 study. Due to this outcome Marinus discontinued this extension study.* *SD - Single Dose, OD - Once daily, BID - Two times Daily, TID - Three times daily, EEG - Electroencephalography, AED - Antiepileptic drug, DB - Double Blind, OL - Open Label, C - Cohort, CDKL5 - Cyclin Dependent Kinase Like 5, CSWS - Continuous Spike and Wave During* *Sleep, M - Male, F - Female*

A	B	C	D	E					
Study	Year	Type of Study	Phase	Dose, mg	Indication	n	Sex, n	Age, Mean (SD)	Race, n (%)
^6^	2000	DB, randomized, placebo controlled clinical trial	-	Day 1 - 500 TIDDay 2 to 8 - 625 TID	Epilepsy	24	M: 16F: 8	37.9 (11.6)	Caucasian: 21 (87.5)Hispanic - 2 (8.3)Arabic - 1 (4.2)
				Placebo	Epilepsy	28	M: 14F: 14	31.9 (9.5)	Caucasian: 20 (71.4)African American: 3 (10.7)Hispanic: 5 (17.9)
^7^	2000	Multicenter, OL, add-on trial	-	Day 1 to 3 - 4.5 mg/kg/dDay 4 to 7 - 9 mg/kg/dWeek 2 - 18 mg/kg/dWeek 3 - 27 mg/kg/dWeek 4 - 36 mg/kg/dFor Next 2 months - Suitable dose for each	Epilepsy	20	M: 11F: 9	2.7	-
^8^	2007	Non-randomized, non-blinded, OL, dose-escalation trial	-	1 mg/kg BID to 12 mg/kg TID	Epilepsy	15	M: 9F: 6	9.2 (3.46)	-
^9^	2017	DB, placebo-controlled,randomized clinical trial	II	Day 1 to 2 - 600/dayDay 3 to 4 - 900/dayDay 5 to 6 - 1,200/dayDay 7 to End of 8 Weeks - 1,500/day	Epilepsy	98	M: 34F: 64	39.1 (11.7)	Caucasian: 87 (88.8)Black/African American: 8 (8.2)Other: 3 (3.0)
				Placebo	Epilepsy	49	M: 13F: 36	40.2 (11.1)	Caucasian: 42 (85.7)Black/African American: 4 (8.2)Other: 3 (6.1)
^10^	2017	DB, crossover-controlled treatmenttrial of ganaxolone	II	Placebo-Ganaxolone9 mg/kg/day to 1800/day	Fragile X Syndrome	29	M: 23F: 6	11.3	Asian: 2Caucasian: 24More than one race: 3
				Ganaxolone-Placebo9 mg/kg/day to 1800/day	Fragile X Syndrome	30	M: 27F: 3	10.6	Asian: 2Black/African American: 4Caucasian: 22More than one race: 2
^11^	2022	DB, OL, randomized, placebo-controlled trial	III	63 mg/kg/day (≤28 kg) or 1800/day (>28 kg)	CDKL5 deficiency disorder	50	M: 11F: 39	Median age, years (IQR): 5∙0 (3.0 to 10.00)	Caucasian: 46 (92)Asian: 2 (4)Other: 2 (4)
				Placebo	CDKL5 deficiency disorder	51	M: 10F: 41	Median age, years (IQR): 7∙0 (4.0 to 11.0)	Caucasian: 47 (92)Asian: 3 (6)Other: 1 (2)
^12^	2022	Multicenter, OL, dose-finding trial	II	500/day (low)650/day (medium)713/day (high)	Epilepsy	17	M: 8F: 9	56.9	-

ganaxolone group. In contrast, the placebo group in Sperling et al.^^13^^ had a mean of 9.2 (30.5) seizures per week. Knight et al.^^7^^ reported a median (IQR) 28-day seizure frequency of 54.0 (31.3 to 147.3) and 49.2 (18.7 to 120.0) in the ganaxolone and placebo groups, respectively.

### Efficacy and Safety

Efficacy of ganaxolone was reported by majority of the trials except^^12,14^^. Across all trials, 26.3% of participants reported >50% reduction from Baseline in Seizure Frequency. According to the trial conducted by Kerrigan et al.^^5^^, the results showed that 33% of participants experienced a reduction in seizure frequency of 25-50% from baseline, while another 33% experienced a reduction of less than 25%, and the remaining 33% experienced a reduction of more than 50%. Similarly, Pieribone et al.^^6^^ reported a 25–49% and <25% reduction in seizure frequency from baseline in 20% and 65% of participants, respectively. In a study conducted by Sperling et al.^^13^^, the mean (SD) percent change in seizure frequency from baseline was -17.6 (48.9) for the ganaxolone group and 2.0 (63.2) for the placebo group. Similarly, results from NCT02519439 also showed percent reduction of mean (SD) -41.86 (44.9) in ganaxolone group. However, this study was discontinued because Ganaxolone missed its primary endpoint in the double-blind portion of the 1042-0603 NCT01963208 study. Trial NCT02358538 investigated ganaxolone as an anti-epileptic drug in Cyclin dependent kinase like 5 (CDKL5), Lennox-Gastaut syndrome, and Protocadherin 19 (PCDH19) patients and reported % Reduction from Baseline in Seizure Frequency as mean (SD) -49.20 (50.2), -37.75 (7.89) and -19.95 (63.57), respectively. They also reported other data for Continuous Spike and Wave During Sleep (CSWS) patients, but patients discontinued from the study so their data for % reduction in seizure frequency is not available. Knight et al.^^7^^ conducted a trial in which CDKL5 deficiency disorder patients were administered both ganaxolone and placebo. The results showed a Median (IQR) Percent Reduction from Baseline in Seizure Frequency of 30.7 (49.5 to 1.9) for ganaxolone and 6.9 (24.1 to ‒39.7) for placebo. The trial NCT03865732^^15^^ reported similar results with the ganaxolone group showing a Median % (IQR) Reduction from Baseline in Seizure Frequency of -61.52 (-95.85 to -33.40), while the placebo group showed a Median % (IQR) Reduction from Baseline in Seizure Frequency of -23.97 (-88.24 to 4.89). Trial NCT00512317^^10^^ also showed a % Change From Baseline in Weekly Seizure Frequency, Mean (SD) of -38.8 (25) for ganaxolone followed by ganaxolone in the extension study and a % Change From Baseline in Weekly Seizure Frequency, Mean (SD) of -43.5 (27.9) for placebo followed by ganaxolone in the extension study. Additionally, Participants who were administered ganaxolone in Cohort 2 of NCT01963208^^16^^ showed a median percent reduction from Baseline in Seizure Frequency of Median (95% CI): -21.28 (-29.60 to -14.29). Meanwhile, those who were given the placebo showed a median percent reduction of Median (95% CI): -10.25 (-20.14 to -1.28). Table 2 presents a detailed overview of the available data on the efficacy of ganaxolone across all studies.

The total treatment-related adverse events (TRAE) occurred in a mean (SD) of 59.95 (29), with 66 (25.3) in the ganaxolone group and 44.3 (33.8) in the placebo group. However, since the sample size of the placebo group was small and the number of studies reporting placebo-controlled data was also limited, the significance of this result may be questionable. The analysis revealed that the nervous system was the most impacted, with dizziness, somnolence, headache, and seizures being the most frequently reported treatment-related adverse events (TRAEs). Other TRAEs, such as decreased appetite, upper respiratory tract infection, drowsiness, and abnormal vocalizations, were only mentioned in one or two trials. Moreover, the mean (SD) for total serious treatment-related adverse events (STRAE) was 11.5 (13.8), with 10.1 (12.4) occurring in the ganaxolone group and 15 (17.6) in the placebo group. Tables 3-5 provide a comprehensive overview of the TRAE associated with ganaxolone treatment and allow for a better understanding of the safety profile of this drug.

Overall, the data suggest that ganaxolone is efficacious in reducing seizure frequency in a variety of disorders, including CDKL5, Lennox-Gastaut syndrome, and PCDH19 patients. Additionally, the study on CDKL5 deficiency disorder patients administered both ganaxolone and placebo showed a moderate reduction in seizure frequency for ganaxolone, but not for placebo. In summary, while ganaxolone appears to be generally efficacious in reducing seizure frequency in various disorders, its effectiveness may vary depending on the specific disorder being treated.

**Table 2:** Outcomes in major clinical trials for Ganaxolone *^*a*^* *: Terminated because ganaxolone missed its primary endpoint in the double-blind portion of the 1042-0603 NCT01963208 study. Due to this outcome Marinus discontinued this extension study.* *SD - Single Dose, OD - Once daily, BID - Two times Daily, TID - Three times daily, EEG - Electroencephalography, AED - Antiepileptic drug, DB - Double Blind, OL - Open Label, C - Cohort, CDKL5 - Cyclin Dependent Kinase Like 5, CSWS - Continuous Spike and Wave During Sleep, M - Male, F - Female, V - Very, M - Much, I - Improved, MN - Minimally, NC - No*

A	B	C	D	E				
Study	Dose, mg	Indication	% Reduction from Baseline in Seizure Frequency	>50% reduction from Baseline in Seizure, n(%)	Clinical Global Impression of Improvement (CGI-I) Scores, n(%)	PGI-I Scores, n(%)	Adverse Events, n(%)	Serious Adverse Events, n(%)
^6^	Day 1 - 500 TIDDay 2 to 8 - 625 TID	Epilepsy	-	-	-	-	19 (79.1)	1 (4.2)
	Placebo	Epilepsy	-	-	-	-	19 (67.8)	1 (3.6)
^7^	Day 1 to 3 - 4.5 mg/kg/dDay 4 to 7 - 9 mg/kg/dWeek 2 - 18 mg/kg/dWeek 3 - 27 mg/kg/dWeek 4 - 36 mg/kg/dFor Next 2 months - Suitable dose for each	Epilepsy	25–50% Decrease - 5(33)<25% Decrease - 5(33)	5 (33)	-	-	14 (73.6)	0
^8^	1 mg/kg BID to 12 mg/kg TID	Epilepsy	25–49% Decrease - 3 (20)<25% Decrease - 13 (65)	4 (27)	-	-	10 (67)	1 (6.7)
^9^	Day 1 to 2 - 600/dayDay 3 to 4 - 900/dayDay 5 to 6 - 1,200/dayDay 7 to End of 8 Weeks - 1,500/day	Epilepsy	Mean (SD): -17.6 (48.9)	23 (23.5)	-	-	82 (83.7)	3 (3)
	Placebo	Epilepsy	Mean (SD): 2.0 (63.2)	7 (14.6)	-	-	38 (77.6)	4 (6.1)
^10^	Placebo-Ganaxolone9 mg/kg/day to 1800/day	Fragile X Syndrome	-	-	Mean (SD): 3.4 (0.13)		24 (83)	0
	Ganaxolone-Placebo9 mg/kg/day to 1800/day	Fragile X Syndrome	-	-	Mean (SD): 3.5 (0.13)		17 (57)	0
^11^	63 mg/kg/day (≤28 kg) or 1800/day (>28 kg)	CDKL5 deficiency disorder	Median (IQR): 30.7 (49.5 to 1.9)	12 (24)	V M I: 0; M I: 7 (15); MN I: 19 (40); NC: 16 (33)MN W: 2 (4); M W: 3 (6); V M W: 1 (2); MN I or better: 26 (54)	V M I: 0; M I: 13 (27); MN I: 17 (35)NC: 14 (29); MN W: 2 (4); M W: 2 (4); V M W: 0; MN I or better: 30 (63)	43 (86)	6 (12)
	Placebo	CDKL5 deficiency disorder	Median (IQR): 6.9 (24.1 to ‒39.7)	5 (10)	V M I: 0; M I: 7 (15); MN I: 13 (27); NC: 19 (40)MN W: 9 (19); M W: 0; V M W: 0MN I or better: 20 (42)	V M I: 1 (2); M I: 7 (15); MN I: 13 (27)NC: 22 (46); MN W: 4 (8); M W: 1 (2); V M W: 0; MN I or better: 21 (44)	45 (88)	5 (10)
^12^	500/day (low)650/day (medium)713/day (high)	Epilepsy	Change in EEG SB after 14 hrs of administration (%):500mg/day (low) - 50%650mg/day (medium) - 70%713mg/day (high) - ≈98%	-	Mean (Range): 4.9 (3–6)		15 (88)	6 (35)

**Table 3:** Adverse Events related to Nervous system *^*a*^* *: Terminated because ganaxolone missed its primary endpoint in the double-blind portion of the 1042-0603 NCT01963208 study. Due to this outcome Marinus discontinued this extension study.* *SD - Single Dose, OD - Once daily, BID - Two times Daily, TID - Three times daily, EEG - Electroencephalography, AED - Antiepileptic drug, DB - Double Blind, OL - Open Label, C - Cohort, CDKL5 - Cyclin Dependent Kinase Like 5, CSWS - Continuous Spike and Wave During Sleep, M - Male, F - Female.*

A	B	C	D	E					
Study	Dose, mg	Indication	Dizziness n (%)	Agitationn (%)	Depression n (%)	Somnolence, n (%)	Convulsions n (%)	Headache, n (%)	Seizures, n (%)
^5^	50-1,500 SD	Epilepsy	14	-	-	12	-	-	-
	≤ 300 BID	Epilepsy	-	-	-	-	-	-	-
	500 OD	Epilepsy	-	-	-	-	-	-	-
^6^	Day 1 - 500 TIDDay 2 to 8 - 625 TID	Epilepsy	4 (16.6)	1 (4.1)	1 (4.1)	-	-	-	-
	Placebo	Epilepsy	3 (10.7)	0	0	-	-	-	-
^7^	Day 1 to 3 - 4.5 mg/kg/dDay 4 to 7 - 9 mg/kg/dWeek 2 - 18 mg/kg/dWeek 3 - 27 mg/kg/dWeek 4 - 36 mg/kg/dFor Next 2 months - Suitable dose for each	Epilepsy	-	-	-	5 (25)	-	-	-
^8^	1 mg/kg BID to 12 mg/kg TID	Epilepsy	-	2 (13.3)	-	9 (60)	3 (20)	-	-
^9^	Day 1 to 2 - 600/dayDay 3 to 4 - 900/dayDay 5 to 6 - 1,200/dayDay 7 to End of 8 Weeks - 1,500/day	Epilepsy	16 (16.3)	-	-	13 (13.3)	5 (5.1)	8 (8.2)	-
	Placebo	Epilepsy	4 (8.2)	-	-	1 (2.0)	4 (8.2)	6 (12.2)	-
^10^	Placebo-Ganaxolone9 mg/kg/day to 1800/day	Fragile X Syndrome	3 (1.6)	4 (2.1)	-	-	-	5 (2.6)	1 (0.5)
	Ganaxolone-Placebo9 mg/kg/day to 1800/day	Fragile X Syndrome	1 (0.7)	7 (4.8)	-	-	-	5 (3.4)	1 (0.7)
^11^	63 mg/kg/day (≤28 kg) or 1800/day (>28 kg)	CDKL5 deficiency disorder	-	-	-	17 (34)	-	-	4 (8)
	Placebo	CDKL5 deficiency disorder	-	-	-	3 (6)	-	-	4 (8)
^13^	50/ml TID	Epilepsy	-	2 (20)	-	4 (40)	-	-	0
	Placebo	Epilepsy	-	0		3 (27.27)			4 (36.36)
^14^	63 mg/kg/day to Maximum 1800/day	Epilepsy in CDKL5	-	-	-	0	-	-	1 (14.29)
		Epilepsy in CSWS	-	-	-	1 (50)	-	-	0
		Epilepsy in Lennox-Gastaut	-	-	-	2 (20)	-	-	2 (20)
		Epilepsy in PCDH19	-	-	-	6 (54.55)	-	-	4 (36.36)
^15^	Ganaxolone in 1042-600 followed by ganaxolone in Extension StudyDay 1 to 2 - 300 TIDDay 2 to 4 - 400 TIDDay 5 to End - 500 TID	Epilepsy	16 (13.01)	-	6 (4.88)	10 (8.13)	20 (16.26)	26 (21.14)	-
	Placebo In 1042-600 followed by ganaxolone in Extension Study	Epilepsy	-	-	-	-	-	-	-

Overall, the results of these trials suggest that ganaxolone may be effective in reducing seizure frequency in patients with various types of epilepsy, including CDKL5 deficiency disorder, Lennox-Gastaut syndrome, and Protocadherin 19. However, further studies are needed to determine its safety and efficacy in a larger patient population.

## DISCUSSION

The purpose of this systematic review was to evaluate the safety and efficacy of ganaxolone, an anti-epileptic drug, using data from phase 2 and 3 randomized controlled trials. The findings indicate that the drug is consistently effective and safe for use in diverse patient populations across all trials. The clinical trials for ganaxolone included patients with various conditions such as Fragile X Syndrome, CDKL5 deficiency disorder, CSWS, Lennox-Gastaut, and PCDH19. These results have clinical implications that can guide clinicians in using ganaxolone to treat different conditions.Overall, ganaxolone has a favorable safety profile, with most treatment-related adverse events (TRAEs) being classified as mild to moderate in severity. While the ganaxolone group had a slightly higher incidence of TRAEs compared to the placebo group, this difference cannot be reliably interpreted due to the small sample size of the placebo group and limited availability of placebo-controlled data. The most commonly reported TRAEs were dizziness, somnolence, headache, and seizures, with the nervous system being the most commonly affected system.Multiple trials have shown that ganaxolone has a significant positive effect on the treatment of seizures and postpartum depression^^17^^. Additionally, evidence suggests that ganaxolone may be effective in treating anxiety disorders and neuropathic pain^^14^^. The magnitude of the treatment effect appears to be moderate to large, and the consistency of results across studies is generally positive. However, patient characteristics such as age and comorbidities, as well as dosing regimen, may influence the efficacy of ganaxolone. Compared to other treatments, ganaxolone appears to have similar or superior efficacy, with fewer side effects. However, the available efficacy data on ganaxolone as an anti-epileptic drug is limited, and further research is needed to fully evaluate its potential in various conditions. Overall, while ganaxolone shows promise as a new treatment option, more research is necessary to fully understand its efficacy and safety.

**Table 4:** Adverse Events related to Gastrointestinal system *^*a*^* *: Terminated because ganaxolone missed its primary endpoint in the double-blind portion of the 1042-0603 NCT01963208 study. Due to this outcome Marinus discontinued this extension study.* *SD - Single Dose, OD - Once daily, BID - Two times Daily, TID - Three times daily, EEG - Electroencephalography, AED - Antiepileptic drug, DB - Double Blind, OL - Open Label, C - Cohort, CDKL5 - Cyclin Dependent Kinase Like 5, CSWS - Continuous Spike and Wave During Sleep, M - Male, F - Female.*

A	B	C	D	E		
Study	Dose, mg	Indication	Diarrhoea, n (%)	Constipation, n (%)	Vomiting, n (%)	Nausea, n (%)
^7^	Day 1 to 3 - 4.5 mg/kg/dDay 4 to 7 - 9 mg/kg/dWeek 2 - 18 mg/kg/dWeek 3 - 27 mg/kg/dWeek 4 - 36 mg/kg/dFor Next 2 months - Suitable dose for each	Epilepsy	4 (20)	3 (15)	3 (15)	-
^8^	1 mg/kg BID to 12 mg/kg TID	Epilepsy	-	1 (6.67)	-	-
^10^	Placebo-Ganaxolone9 mg/kg/day to 1800/day	Fragile X Syndrome	10 (5.3)		7 (3.7)	1 (0.5)
	Ganaxolone-Placebo9 mg/kg/day to 1800/day	Fragile X Syndrome	10 (6.9)		7 (4.8)	1 (0.7)
^11^	63 mg/kg/day (≤28 kg) or 1800/day (>28 kg)	CDKL5 deficiency disorder	-	3 (6)	-	-
	Placebo	CDKL5 deficiency disorder	-	0	-	-
^13^	50/ml TID	Epilepsy	1 (10)	-	0	-
	Placebo	Epilepsy	2 (18.18)	-	2 (18.18)	-
^14^	63 mg/kg/day to Maximum 1800/day	Epilepsy in CDKL5	-	-	3 (42.86)	-
		Epilepsy in CSWS	-	-	1 (50)	-
		Epilepsy in Lennox-Gastaut	-	-	1 (10)	-
		Epilepsy in PCDH19	-	-	3 (27.27)	-
^15^	Ganaxolone in 1042-600 followed by ganaxolone in Extension StudyDay 1 to 2 - 300 TIDDay 2 to 4 - 400 TIDDay 5 to End - 500 TID	Epilepsy	5 (4.07)	-	6 (4.88)	12 (9.76)
	Placebo In 1042-600 followed by ganaxolone in Extension Study	Epilepsy	-	-	-	-
^17^ ^a^	450 to 900 BID; max. 1800/day	Epilepsy	-	-	-	1 (3.85)

## CONCLUSION

Various studies have demonstrated that ganaxolone is an effective treatment for seizures, with a moderate to large positive impact. The results of these studies have been consistently positive, with ganaxolone showing similar or better efficacy compared to other treatments, while producing fewer side effects. However, the data on ganaxolone's effectiveness as an anti-epileptic drug is limited, and additional research is required to evaluate its potential for treating various conditions. In summary, while ganaxolone presents a promising new option for treating seizures, further research is necessary to fully comprehend its efficacy and safety.

**Table 5:** Adverse Events related to other systems. *^*a*^* *: Terminated because ganaxolone missed its primary endpoint in the double-blind portion of the 1042-0603 NCT01963208 study. Due to this outcome Marinus discontinued this extension study.* *SD - Single Dose, OD - Once daily, BID - Two times Daily, TID - Three times daily, EEG - Electroencephalography, AED - Antiepileptic drug, DB - Double Blind, OL - Open Label, C - Cohort, CDKL5 - Cyclin Dependent Kinase Like 5, CSWS - Continuous Spike and Wave During Sleep, M - Male, F - Female.*

A	B	C	D	E				
Study	Dose, mg	Indication	Pyrexia, n (%)	Rash, n (%)	Fatigue, n (%)	Fall, n (%)	Rhinitis, n (%)	Nasopharyngitis, n (%)
^7^	Day 1 to 3 - 4.5 mg/kg/dDay 4 to 7 - 9 mg/kg/dWeek 2 - 18 mg/kg/dWeek 3 - 27 mg/kg/dWeek 4 - 36 mg/kg/dFor Next 2 months - Suitable dose for each	Epilepsy	-	1 (5)	-	-	1 (5)	-
^9^	Day 1 to 2 - 600/dayDay 3 to 4 - 900/dayDay 5 to 6 - 1,200/dayDay 7 to End of 8 Weeks - 1,500/day	Epilepsy	-	-	16 (16.3)	5 (5.1)	-	5 (5.1)
	Placebo	Epilepsy	-	-	4 (8.2)	6 (12.2)	-	5 (10.2)
^10^	Placebo-Ganaxolone9 mg/kg/day to 1800/day	Fragile X Syndrome		9 (4.8)	28 (14.8)	2 (1.1)	1 (0.5)	
	Ganaxolone-Placebo9 mg/kg/day to 1800/day	Fragile X Syndrome		6 (4.1)	16 (11.0)	1 (0.7)	3 (2.1)	
^13^	50/ml TID	Epilepsy	-	0	2 (20)	-	-	-
	Placebo	Epilepsy	-	2 (18.18)	2 (18.18)	-	-	-
^14^	63 mg/kg/day to Maximum 1800/day	Epilepsy in CDKL5	5 (71.43)	1 (14.29)	-	-	-	-
		Epilepsy in CSWS	0	0	-	-	-	-
		Epilepsy in Lennox-Gastaut	1 (10)	1 (10)	-	-	-	-
		Epilepsy in PCDH19	7 (63.64)	1 (9.09)	-	-	-	-
^15^	Ganaxolone in 1042-600 followed by ganaxolone in Extension StudyDay 1 to 2 - 300 TIDDay 2 to 4 - 400 TIDDay 5 to End - 500 TID	Epilepsy	5 (4.07)	7 (5.69)	20 (16.26)	17 (13.82)	-	17 (13.82)
	Placebo In 1042-600 followed by ganaxolone in Extension Study	Epilepsy	-	-	-	-	-	-
^16^	DB: C 1 - Ganaxolone - 1200/day and 1800/day + AED	Epilepsy	-	-	2 (8.33)	-	-	0
	DB: C 1 - Placebo - Placebo + AED	Epilepsy	-	-	0	-	-	0
	DB: C 2 - Ganaxolone - 1800/day + AED	Epilepsy	-	-	21 (11.73)	-	-	5 (2.79)
	DB: C 2 - Placebo - Placebo + AED	Epilepsy	-	-	12 (6.82)	-	-	9 (5.11)
	OL: Ganaxolone in Double-blind Phase - 1800/day + AED	Epilepsy	-	-	9 (5.70)	-	-	6 (3.80)
	OL: Placebo in Double-blind Phase - Ganaxolone + AED	Epilepsy	-	-	15 (8.67)	-	-	9 (5.20)
^17^ ^a^	450 to 900 BID; max. 1800/day	Epilepsy	1 (3.85)	-	-	1 (3.85)	-	1 (3.85)
